# Atypical Clostridium difficile Infection in a Pregnant Patient: A Case Study on Non-Diarrheal Presentation and Syndrome of Inappropriate Antidiuretic Hormone (SIADH) Complication

**DOI:** 10.7759/cureus.53449

**Published:** 2024-02-02

**Authors:** Mohamed Ismail, Ritik Goyal, Menna-Allah A Elaskandrany, Michael Bebawy, Sahiba Singh, Claire Ruane, Weizheng Wang

**Affiliations:** 1 Department of Medicine, Rutgers University New Jersey Medical School, Newark, USA; 2 Internal Medicine, Lenox Hill Hospital, Manhattan, USA; 3 Medicine, College of Osteopathic Medicine, Michigan State University, East Lansing, USA; 4 Department of Internal Medicine and Pediatrics, Rutgers University New Jersey Medical School, Newark, USA; 5 Gastroenterology and Hepatology, Rutgers University New Jersey Medical School, Newark, USA

**Keywords:** nonspecific abdominal pain, euvolemic hyponatremia, syndrome of inappropriate secretion of antidiuretic hormone (siadh), young female with constipation, c diff

## Abstract

*Clostridium difficile* (*C. difficile*) is a Gram-positive, spore-producing bacterium that often leads to pseudomembranous colitis, typically manifesting as watery diarrhea. The risk factors for *C. difficile* infection (CDI) include exposure to broad-spectrum antibiotics, immunocompromised states, advanced age, usage of proton pump inhibitors (PPI), and comorbid conditions such as chronic kidney disease (CKD). This report details a case involving a 23-year-old pregnant woman who presented with symptoms of abdominal pain and constipation. She was diagnosed with a urinary tract infection (UTI) and treated with ceftriaxone. During her hospitalization, she was administered opioid pain relievers and underwent an intensive bowel regimen. Despite these measures, her constipation and abdominal discomfort persisted, and magnetic resonance imaging (MRI) of the abdomen revealed significant dilatation of the large bowel. The patient, discovered to have hyponatremia, underwent further evaluation. This revealed elevated urine osmolality and decreased blood plasma osmolality, indicative of a syndrome of inappropriate antidiuretic hormone secretion (SIADH). The patient received treatment with hypertonic saline. Later in her hospital stay, she tested positive for CDI through stool analysis and was treated with oral vancomycin. This case underscores the importance of considering CDI as a differential diagnosis in cases of ileus, abdominal pain, and constipation, especially in patients with notable risk factors for CDI. It highlights that the presence of diarrhea or watery bowel movements is not a necessary symptom for CDI testing.

## Introduction

*Clostridium difficile*, a Gram-positive, spore-forming bacterium, is a well-recognized cause of pseudomembranous colitis, typically presenting with watery diarrhea. It synthesizes toxins A and B, which lead to significant mucosal damage and the formation of pseudomembranous colitis [1]. Key risk factors for *C. difficile* infection (CDI) include disruptions in the normal gut microbiota due to the use of broad-spectrum systemic antibiotics, with clindamycin, cephalosporins, and fluoroquinolones being the most frequently implicated. Additionally, reduced immune function, as seen in elderly individuals (over 65 years) or those with medical comorbidities, the usage of proton pump inhibitors (PPI), and the presence of chronic kidney disease (CKD) also contribute to increased susceptibility to CDI [1-3].

The Society for Healthcare Epidemiology of America and the Infectious Diseases Society of America recommend a comprehensive approach for diagnosing CDI that encompasses both clinical symptoms and laboratory data. A definitive diagnosis usually requires two main criteria: first, the observation of clinical symptoms, particularly diarrhea defined as the passage of three or more loose stools in less than 24 hours; and second, a positive stool test result confirming the presence of toxigenic *C. difficile* or its toxins. Additionally, evidence of pseudomembranous colitis obtained through colonoscopy or histopathology can further substantiate the diagnosis [4,5].

CDI can be categorized into four types: mild to moderate, severe, severe complicated, and recurrent CDI. Mild to moderate CDI is characterized by diarrhea without systemic infection signs, a WBC count below 15,000, and serum creatinine levels not exceeding 1.5 times the baseline. Severe CDI is identified by the presence of systemic infection indicators such as fever, a WBC count above 15,000, or serum creatinine levels more than 1.5 times the baseline. Severe complicated CDI includes symptoms of hypotension, ileus, or megacolon, in addition to the criteria for severe CDI. CDI is considered recurrent if it reappears within 8 weeks following the successful completion of initial treatment [3].

While diarrhea is the most common symptom in patients with CDI, other symptoms such as abdominal pain, fever, and leukocytosis occur in less than half of these cases. Instances of CDI without diarrhea, although not rare in clinical settings, particularly among critically ill patients, are infrequently documented in medical literature, likely due to a lack of case reporting [5].

We present a unique case of CDI in a pregnant woman, manifesting with atypical symptoms of constipation rather than the more common presentation of watery diarrhea. This case is further complicated by the development of hyponatremia, which, upon investigation, appears to be most likely attributed to the syndrome of inappropriate antidiuretic hormone secretion (SIADH). This case adds a significant dimension to the understanding of CDI, particularly in the context of pregnancy, constipation, and SIADH, emphasizing the need for further research in this domain.

## Case presentation

A 23-year-old female, gravida 3, para 2, with no notable past medical history, was admitted to a neighboring hospital following 5 days of persistent abdominal pain and constipation. Two days before her current hospital admission, she had sought medical attention at the emergency department for similar symptoms. At that time, a urinalysis indicated an infection, leading to a prescription of amoxicillin 500 mg to be taken every 8 hours for a duration of 5 days, after which she was discharged. However, the patient returned to the hospital as her symptoms remained unresolved. This abdominal pain was along with nausea and four instances of non-bilious, non-bloody vomiting within a span of one day. The abdominal pain, initially mild, worsened to severe, particularly in the lower abdomen, extending to the back and right flank. The patient experienced chills, but no fever, and her vital signs were stable upon admission. Physical examination revealed tenderness in the epigastric area and both right and left lower quadrants without guarding or rebound tenderness. Neither Rovsing's nor McBurney's signs were present, but costovertebral angle tenderness was noted. Laboratory findings, including a complete blood count and metabolic panel, were within normal limits, although urinalysis indicated a potential infection. Abdominal ultrasound showed a 5-week gestational sac and fullness in the right renal system. She was hospitalized and started on intravenous ceftriaxone 1 g IV for 5 days for presumed pyelonephritis and morphine 2.5 mg every 4 hours as needed for pain management.

Her hospitalization was complicated by continuous vomiting, severe hyponatremia (sodium level at 120 mEq/L) (Table 1), and constipation from the second day. She was admitted to the ICU for intensive management. Laboratory findings revealed a reduced serum osmolality of 248 mosm/kg, alongside urine osmolality and urine sodium levels of 363 mOsm/kg and 131 mOsm/kg, respectively (Table 1). The patient was found to be euvolemic. After ruling out other potential causes, such as diuretic use, renal disease, hypothyroidism, and adrenal insufficiency (Table 1), these results collectively supported a diagnosis of SIADH. Management included a bowel regimen and 3% hypertonic saline for the symptomatic hyponatremia. Although her sodium levels improved with hypertonic saline, they remained slightly below the normal range (130 mEq/L). Ceftriaxone was discontinued after 5 days.

**Table 1:** Laboratory values on admission. *The sample was taken at 8 am. TSH: thyroid-stimulating hormone

Investigations	Patient values	Reference range
Serum sodium	120 mEq/L	133-145 mEq/L
Serum glucose random	99 mg/dL	70-109 mg/dL
Serum creatinine	0.6 mg/dL	0.74-1.35 mg/dL
Serum osmolality	248 mOsm/kg	275-295 mOsm/kg
TSH	0.25 uIU/mL	0.27-4 uIU/mL
Cortisol level	19.5 ug/dL*	6.20-19.40 ug/dL
Urine osmolality	363 mOsm/kg	300-1000 mOsm/kg
Urine sodium random	131 mEq/L	20 mEq/L

**Figure 1:**
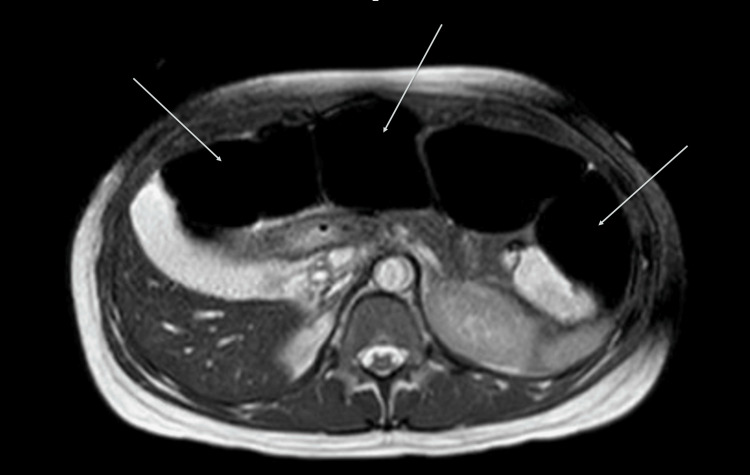
MRI of the abdomen without intravenous (IV) contrast. Moderate to severe dilation of the transverse colon and right colon with a change of caliber at the level of the proximal descending colon.

Despite treatment, the patient’s abdominal pain and constipation persisted, necessitating an expanded bowel regimen including polyethylene glycol 17 g per oral daily, sennosides 17.2 mg per oral daily, and discontinuation of morphine. An abdominal X-ray was deferred due to her pregnancy, and an MRI revealed moderate to severe dilatation in the transverse and right colon, with a caliber change at the proximal descending colon (Figure 1). The patient's constipation remained unresponsive to laxatives. On the 5th day, a tap water enema successfully induced a bowel movement. Additionally, for pain management, dicyclomine 10 mg was prescribed, to be taken orally every 6 hours as required.

On the 9th day, she was transferred to our hospital for further management and potential pregnancy termination. The decision to terminate the pregnancy was in accordance with the patient's wishes, as it was an undesired pregnancy. She experienced widespread body pain, and pain management was resumed with morphine 2.5 mg every 4 hours as needed. The bowel regimen continued with polyethylene glycol 17 g per oral daily, sennosides 17.2 mg per oral daily, and lactulose 20 g per oral daily.

The differential diagnoses for her constipation at this point included opioid-induced ileus, dehydration-related constipation, colitis, and large bowel obstruction. The patient was transitioned to a clear liquid diet and weaned off opioids. A stool examination for *C. difficile*, conducted in response to her prolonged hospitalization and use of antibiotics, revealed a positive outcome. This involved PCR testing for *C. difficile* toxin genes, followed by antigen assays for toxins A and B. She was started on oral vancomycin 125 mg q6h for 10 days. By the 16th day of her total hospital stay, her electrolyte levels had returned to normal. Subsequently, she was discharged to continue her vancomycin treatment at home. Additionally, an elective termination of her pregnancy was arranged on an outpatient basis.

Her abdominal pain, constipation, and body aches resolved around day 7 of oral vancomycin treatment, and she completed the 10-day course of antibiotics. One month post-discharge, she was readmitted to another hospital for a spontaneous miscarriage. A 3-month follow-up call confirmed no symptoms since her discharge from the hospital.

## Discussion

The administration of broad-spectrum antibiotics significantly increases the risk of CDI, with an 8-10-fold heightened risk during and up to 4 weeks following antimicrobial therapy [6]. In the presented case, the patient was treated with ceftriaxone for a urinary tract infection (UTI). Furthermore, hospitalization is a notable risk factor for *C. difficile* colonization and subsequent CDI. At the onset of hospitalization, colonization rates range from 2.1% to 20%, escalating to as much as 45.4% with prolonged stays [6]. Opioid analgesics, used here for pain management, also contribute to an increased CDI risk, potentially due to enhanced *C. difficile* interaction with the mucosa caused by the slowed intestinal transit. This delay may increase intraluminal toxin concentrations, thereby elevating CDI risk [7].

Although pregnant women were historically considered low risk due to their younger age and overall health, physiological and immunological changes in pregnancy could increase their susceptibility. The “Th2 phenomena” of pregnancy, which reduces fetal rejection and miscarriage, also lowers immunoglobulin G antibodies against toxins A and B, typically produced by the Th1 system. This could make pregnant women more vulnerable to CDI. Furthermore, studies suggest that pregnant women may experience a more severe course of CDI than non-pregnant women [8].

Although diarrhea is a common symptom of CDI, it is important to consider CDI in patients with ileus and multiple risk factors. A case involving a 66-year-old man with *C. difficile* colitis presented with ileus, characterized by abdominal pain, constipation, distension, and hyponatremia, diagnosed via colonoscopy and stool testing [9]. Another case involved a 70-year-old woman with CDI, exhibiting neither diarrhea nor abdominal pain [5]. In a series of nine patients with CDI, only one experienced watery stool, while the others presented with constipation or abdominal distension [10].

Hyponatremia, a known complication of CDI, is often secondary to diarrhea-induced fluid imbalances [11]. Interestingly, some studies have also reported cases of *C. difficile* leading to pseudomembranous colitis and presenting with hyponatremia, even in the absence of diarrhea [12]. In the case we present, the patient presented with constipation and hyponatremia. Despite an abdominal MRI showing no definitive signs of colonic inflammation, a diagnosis of pseudomembranous colitis could not be conclusively ruled out as a colonoscopy was deferred due to the patient's pregnancy.

The etiology of hyponatremia was investigated through urine and blood tests, suggesting SIADH as the most probable cause. However, common etiologies typically associated with SIADH, such as medications, malignancy, central nervous system disturbances, surgery, hormone deficiencies or administration, pulmonary diseases, and HIV, were all excluded in this patient [13].

It is crucial to include acute intermittent porphyria (AIP) in the case report as a possible underlying cause for SIADH, given its potential to present with symptoms similar to those experienced by our patient. This underscores the necessity for medical professionals to consider AIP in cases where symptoms continue despite addressing typical causes. This is in line with a prior case report involving *C. difficile*, in which abdominal pain persisted even after the infection had been treated and the patient was found to have AIP. Such instances highlight the frequent oversight of AIP and underscore the importance of a thorough differential diagnosis process in cases presenting with acute abdominal pain, new onset of ileus, and electrolyte imbalances. Specifically, AIP should be taken into account when abdominal pain does not subside, even after treating underlying conditions and ruling out more prevalent etiologies [14].

Current literature establishes a link between SIADH and systemic strongyloidiasis, with the underlying mechanisms remaining to be elucidated [15]. This reference to systemic strongyloidiasis, another gastrointestinal infection, is utilized to draw comparisons with *C. difficile*, also a gastrointestinal infection. However, the association between SIADH and CDIs has not been thoroughly explored. This particular case contributes a novel viewpoint to the spectrum of *C. difficile* complications and underscores the need for further investigative research in this area.

## Conclusions

This case highlights the critical importance of considering CDI in patients presenting with symptoms like abdominal distension, pain, or constipation, particularly when they have been exposed to broad-spectrum antibiotics, hospitalizations, or opioid analgesics, even if they do not exhibit pronounced diarrhea. The patient's condition was complicated by recent antibiotic use and opioid exposure, both of which are significant risk factors for CDI. Moreover, her pregnancy, traditionally considered low-risk for CDI, may actually have increased her susceptibility due to immunological changes associated with gestation.

Her atypical presentation, characterized by constipation and hyponatremia instead of classic diarrhea, underscores the need for a broadened clinical vigilance for CDI. This case advocates for the necessity of *C. difficile* testing beyond the conventional symptomatology of watery stools or diarrhea, especially in patients with significant risk factors. Early detection of CDI is imperative to prevent severe complications, such as fulminant colitis or toxic megacolon.

Furthermore, the investigation into the patient's hyponatremia led to a diagnosis of SIADH, and the consideration of AIP as a potential underlying cause added another layer of complexity to the case. This patient’s clinical course illustrates the importance of a comprehensive differential diagnosis in complex cases, particularly when symptoms persist despite initial treatment strategies.

Overall, this case not only emphasizes the importance of considering CDI in the differential diagnosis of patients with atypical gastrointestinal symptoms and significant risk factors but also highlights the necessity for ongoing research into the associations between gastrointestinal infections like *C. difficile* and systemic conditions such as SIADH and AIP. Such insights are crucial for enhancing our understanding and management of these complex medical conditions in a diverse patient population.
